# The 7th Barossa Meeting—Cell Signalling in Cancer Biology and Therapy in Barossa Valley, Australia

**DOI:** 10.1038/cddis.2016.39

**Published:** 2016-03-03

**Authors:** M A Grimbaldeston, V M Dixit, M S Samuel

**Affiliations:** 1 Centre for Cancer Biology, University of South Australia and SA Pathology, IMVS Building, Frome Road, Adelaide, SA 5000, Australia; 2 Department of Physiological Chemistry, Genentech Inc., South San Francisco, CA 94080, USA

## Cancer cell signalling networks

The 7th Barossa Meeting ‘Cell Signalling in Cancer Biology and Therapy' organised by the Centre for Cancer Biology, Adelaide, brought together an outstanding array of international leaders in cell signalling systems investigating novel therapeutic approaches in cancer.

John Scott (University of Washington, USA) examined how the location of signalling complexes is regulated at the angstrom level by anchoring proteins such as the A-kinase anchor proteins (AKAPs). Using a combination of cryo-electron microscopy and super-resolution microscopy approaches, he demonstrated that manipulating the AKAP12/Aurora A/PLK1 complex causes mitotic delay as a result of inappropriate assembly of astral microtubules causing misorientation of the spindle, thereby revealing the importance of spatial anchoring of this particular signalling complex to symmetric cell division.

Katharina Gaus (University of New South Wales, Australia) continued the theme of spatial control of signalling complexes. She illustrated using super-resolution single-molecule localisation microscopy how T lymphocytes avoid the problem of constant activation of their T-cell antigen receptor via the constitutive kinase Lck, by only becoming activatable upon clustering. Gaus demonstrated that signalling capacity is determined by the density of the clusters, with dense clusters being triggered for signalling and non-dense clusters remaining untriggered. Clustering density depended on the dose of MHC-bound antigen peptide bound to the cluster, a discovery facilitated by super-resolution microscopy that would have been virtually impossible using standard biochemical approaches.

## Translational medicine

Basic research advances provide the essential foundation for translating scientific knowledge into new therapies. In the last two decades, it has emerged that many stem cell lineages retain the ability to renew and produce specialised cells. The existence of ‘cancer stem cells' is controversial as they represent rare and heterogeneous populations that are difficult to identify. Targeting such cells therapeutically without also destroying normal stem cells has been challenging. Lgr5+ cells of the intestinal crypt are epithelial stem cells that may also be a cell of origin for intestinal tumours. Fred de Sauvage (Genentech Inc., USA) elegantly demonstrated using a Lgr5^DTR^ mouse model that ablation of Lgr5+ cells caused irreversible crypt loss and deterioration of intestinal architecture in the context of radiation-induced intestinal damage. De Sauvage's findings also suggest that Lgr5+ stem cells might have features that distinguish them from other stem cell populations, enabling their selective targeting in the intestine, which could offer significant therapeutic promise in a subset of colorectal cancers.

Vishva Dixit (Genentech Inc., USA), a member of the Clifford Prize alumni (2011 recipient), provided new insights into the complex biological process of inflammasome activation to combat bacterial infections. How caspase-11 that is directly activated by intracellular LPS from Gram-negative bacteria executes downstream signalling events that cause pyroptotic cell death, interleukin 1-β processing and lethal septic shock was largely unknown. Dixit's team made the exciting discovery of a new non-canonical mechanism of inflammasome signalling in which caspase-11 (human caspase-4) cleaves gasdermin d to produce an amino-terminal fragment that triggers two distinct cell-intrinsic signals: pyroptosis induction and NLRP3-dependent caspase-1 activation. The pathological importance of these unexpected findings is evident in gasdermin D-deficient mice that are resistant to lethal endotoxemia, thus demonstrating that gasdermin D is a critical component of antibacterial responses.

## Cancer genomics

In one of the most engaging talks of the conference, KJ Patel (Cambridge, UK) addressed how the genome is protected against cancer-causing mutations. Using genetic mouse models and informative alcohol-dosage regimes, Patel revealed the importance of acetaldehyde and formaldehyde clearance to the maintenance of genomic integrity. These two toxins are produced when alcohol is metabolised, and are highly genotoxic, requiring the function of aldehyde dehydrogenase 2 (ALDH2) and alcohol dehydrogenase 5, respectively, for their clearance. Alcohol was highly teratogenic in mice lacking the *ALDH2* gene and the *FANCD2* gene (a causal gene for Fanconi anaemia, a myelodysplastic cancer syndrome) and surviving mice spontaneously developed acute myeloid leukaemia. Given the frequency with which *ALDH2* gene mutations occur in human populations, particularly of East Asian descent, these data suggest a new mechanism by which alcohol consumption elevates cancer risk.

## Cancer signalling architecture

**Figure 1:**
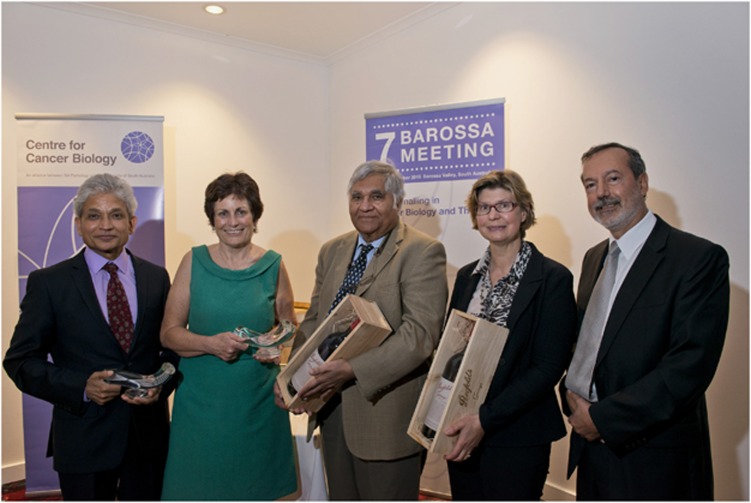
The Clifford Prize for Cancer Research, awarded at each Barossa Meeting, was presented to Professor Inder Verma and Professor Jane Visvader. Left to right: Professor Sharad Kumar (Centre for Cancer Biology, Adelaide), Dr. Leanna Read (Chief Scientist, South Australia), Professor Inder Verma (The Salk Institute, USA), Professor Jane Visvader (The Walter and Eliza Hall Institute of Medical Research, Australia) and Professor Angel Lopez (Centre for Cancer Biology, Adelaide)

Breast cancers are highly heterogeneous and can arise from breast-epithelial stem cells and progenitor cells. Understanding the normal breast epithelial hierarchy would therefore be instructive in understanding breast cancer heterogeneity. Jane Visvader (Melbourne, VIC, Australia), corecipient of the 2015 Clifford Prize for Cancer Research (Figure 1), discussed her recent advances in dissecting the normal mammary epithelial hierarchy, taking advantage of the confetti mouse and a three-dimensional imaging approach to reveal that cytokeratin 5-positive progenitor cells contribute to both the basal and luminal lineages. Elf5-positive progenitor cells on the other hand contribute solely to the luminal lineage. Cytokeratin 5-positive progenitors also contribute to alveologenesis. Interestingly, three-dimensional imaging also uncovered a novel population of alveolar cells primed for milk production in lactating mammary glands.

## Cancer cell biology

An emerging theme in cancer biology is the role for ubiquitination in autophagy. The role of autophagy in cancer and immune pathologies is complex and context dependent, as highlighted by the presentations of Ivan Dikic (Goethe University, Germany) and Junying Yuan (Harvard Medical School, USA). Dikic has identified that UBAN ubiquitin binding domain is present in ABIN, NEMO and OPTN proteins, which are implicated in NFκB activation and autophagy. Sharpin, a novel component of the linear ubiquitin assembly complex (LUBAC), is thought to be required for linear ubiquitination of NEMO, activating the inhibitors of κB kinases (IKKs) in the NFκB pathway and apoptotic pathways downstream of TNF receptors. Of note, Dikic indicated that linear ubiquitination is involved in cellular resistance to chemotherapeutics as mutations in HOIP (component of LUBAC), that are linked to aggressive forms of leukaemia, can be blocked by specific inhibitors of LUBAC action.

Junying Yuan then proposed the notion of activating chaperone-mediated autophagy (CMA), a subtype of autophagy that delivers selected proteins into the lysosome for degradation, as a potential anticancer therapy. Although inhibition of autophagy itself is not sufficient to induce cancer cell death, Yuan's team demonstrated that inhibition of the receptor tyrosine kinase FLT3 signalling in non-hematopoietic cancers renders them sensitive to CMA inhibition. Simultaneous inhibition of CMA and targeting of FLT3 with the specific inhibitor AC220 leads to death of cancer cells via a mechanism involving the degradative loss of the glycolytic enzyme hexokinase 2. These findings potentially delineate a novel strategy to promote the death of cancer cells that have acquired mutations to resist intrinsic pathways of apoptosis through metabolic catastrophe.

## Cancer cell signalling systems

In this final session of the meeting, Richard Marais (Cancer Research UK Manchester Institute, UK) continued the theme of signalling networks in melanomagenesis. Marais has developed a powerful technology platform centred on patient-derived melanoma xenografts to test the responses of BRAF mutant tumours to targeted therapies. The protocol also entails whole-exome sequencing and targeted sequencing of circulating tumour DNA from patients with advanced melanoma to monitor responses to therapy and identify mechanisms of resistance, which in turn is validated in the patient-derived xenografts. This platform can provide timely information to clinicians, enabling individualised precision therapy for patients.

Inder Verma (The Salk Institute, USA), corecipient of the 2015 Clifford Prize for Cancer Research (Figure 1), concluded the meeting. To cause oncogenic changes in the context of normal cells, Verma introduced lentiviruses encoding two conditional oncogenes into the cortex, hippocampus and subventricular zone of the murine brain, generating invasive tumours. The tumours were serially transplantable, suggesting that cancer stem cells were present. Early tumours expressed markers of the three major CNS lineages and progenitor markers. However, as tumours progressed, CNS markers were attenuated and Nestin, a marker of neural progenitors, was progressively elevated. Verma proposed that oncogenic transformation of differentiated glia causes dedifferentiation to produce stem-like cells, thereby yielding the heterogeneous cell population observed in glioma.

In conclusion, it is clear that significant progress has been made in understanding the complexities in cell signalling while much remains to be discovered, as therapeutic intervention moves toward a precision approach. We look forward to the next Barossa Cell Signalling meeting in 2017, with anticipation that it will continue to provide an important international forum for high-level scientific discourse on emerging insights into cell signalling and therapeutic advances in cancer.

